# Utilidad del *score* H_2_FPEF en el diagnóstico y pronóstico de las insuficiencia cardiaca con fracción de eyección preservada en atención primaria

**DOI:** 10.1016/j.aprim.2025.103335

**Published:** 2025-10-07

**Authors:** Victoria Cendrós, José María Verdú-Rotellar, Jaume Parra, Miguel Ángel Muñoz, Mar Domingo, Josep Franch

**Affiliations:** a Fundació Institut Universitari per a la recerca a l’Atenció Primària de Salut Jordi Gol i Gurina (IDIAPJGol), Barcelona, España; b Departamento de Medicina, Universitat de Barcelona, Barcelona, España; c Unidad de Insuficiencia Cardíaca, Departamento de Cardiología, Hospital Universitari Germans Trias i Pujol, Badalona, Barcelona, España; d Centro de Investigación Biomédica en Enfermedades Cardiovasculares (CIBERCV), Barcelona, España; e Department of Medicine and Life Sciences, Pompeu Fabra University, Barcelona, España

**Keywords:** Escala H_2_FPEF, Insuficiencia cardiaca, Fracción de eyección, Atención primaria, H_2_FPEF score, Heart failure, Ejection fraction, Primary care

## Abstract

**Objetivo:**

Determinar, en un grupo de pacientes con diagnóstico de insuficiencia cardiaca (IC) en atención primaria, la probabilidad de presentar insuficiencia cardiaca con fracción de eyección preservada (ICFEP) según el *score* H_2_FPEF y su asociación con la morbimortalidad a un año.

**Diseño:**

Estudio descriptivo transversal.

**Emplazamiento:**

Siete centros de atención primaria de Barcelona.

**Participantes:**

Pacientes con diagnóstico de IC (CIE-10: I.50) y fracción de eyección ≥ 50%.

**Intervenciones:**

Análisis de datos de la historia clínica informatizada en atención primaria y entrevista clínica con el paciente.

**Mediciones principales:**

Se recogieron variables clínicas, analíticas y ecocardiográficas. Se calculó el *score* H_2_FPEF y se clasificó a los pacientes según probabilidad. Se analizó la asociación entre el *score* y el evento combinado (mortalidad o ingreso por IC) al año del cálculo del *score*.

**Resultados:**

Se incluyeron 545 pacientes, en 169 pacientes se dispuso de todas las variables para calcular el *score* H_2_FPEF. De estos, el 47,9% presentó alta probabilidad de ICFEP. Estos pacientes eran mayores, con más comorbilidad y peor clase funcional. La incidencia del evento combinado fue mayor en el grupo de alta probabilidad (64,4%) frente a baja/intermedia (35,6%; p = 0,011). En el análisis multivariado, una alta probabilidad se asoció significativamente con el evento combinado (OR: 2,25; IC 95%: 1,08-4,78; p = 0,031).

**Conclusiones:**

El *score* H_2_FPEF es una herramienta útil y viable en atención primaria para mejorar el diagnóstico y estratificar la morbimortalidad en los pacientes con sospecha de IC.

## Abstract (en)

**Objective:**

To determine, in a group of patients with a diagnosis of heart failure (HF) in primary care, the probability of presenting heart failure with preserved ejection fraction (HFpEF) according to the H_2_FPEF score and its association with one-year morbidity and mortality.

**Design:**

Cross-sectional descriptive study.

**Setting:**

Seven primary care centres in Barcelona.

**Participants:**

Patients with HF (ICD-10: I.50) and left ventricular ejection fraction ≥ 50%.

**Interventions:**

analysis of electronic health record data from primary care and clinical interview with patients.

**Main measurements:**

Clinical, laboratory and echocardiographic variables were collected. The H_2_FPEF score was calculated and patients were classified accordingly. The association between the score and the composite outcome (all-cause mortality or HF hospitalisation) at one year was analysed.

**Results:**

A total of 628 patients were included, of whom 169 had complete data for score calculation. Of these, 47.9% had a high probability of HFpEF. These patients were older, with more comorbidities and worse functional class. The incidence of the composite event was higher in the high-probability group (64.4%) compared to the low/intermediate group (35.6%; *P*=.011). In multivariate analysis, high probability showed a trend towards association with the composite outcome (OR: 2.06; 95% CI: 0.96-4.54; *P*=.066).

**Conclusions:**

The H_2_FPEF score is a useful and feasible tool in primary care for improving the diagnosis and risk stratification of morbidity and mortality in patients with suspected HF.

## Introducción

La insuficiencia cardiaca (IC) es una enfermedad crónica con una incidencia y prevalencia crecientes debido a la mayor expectativa de vida de la población general, y al aumento en la supervivencia de los pacientes afectos de cardiopatía isquémica. Los datos epidemiológicos de la IC la sitúan como un importante problema de salud pública^1^, ^2^, ^3^. Es la tercera causa de muerte cardiovascular en nuestro país con una prevalencia global del 6,8%, aumenta exponencialmente a partir de los 75 años, y es la primera causa de hospitalización en las personas mayores de 65 años^4^, ^5^, ^6^. Se trata de una enfermedad de mal pronóstico, con una tasa de supervivencia más baja que la de determinados cánceres^1^, ^2^, ^3^, ^4^, ^5^. La IC con fracción de eyección preservada (ICFEP) tiene una elevada prevalencia y mal pronóstico. Es responsable de la mitad de los ingresos por descompensación de IC, y presenta una mortalidad equiparable a la ICFER. La definición de insuficiencia cardíaca con ICFEP ha evolucionado desde un diagnóstico de exclusión basado en la clínica, hasta definiciones centradas en la evidencia objetiva de disfunción diastólica y/o presiones de llenado del ventrículo izquierdo elevadas. A pesar de los avances en la comprensión de la fisiopatología de la ICFEP y el desarrollo de modalidades de imagen más sofisticadas, el diagnóstico de ICFEP sigue siendo un reto, especialmente en el entorno de los pacientes crónicos, dado que los síntomas son provocados por el esfuerzo y la evaluación diagnóstica se realiza principalmente en reposo. El estudio hemodinámico invasivo y, en particular, la prueba de esfuerzo invasiva, se considera el método de referencia para el diagnóstico de la ICFEP^7^. Sin embargo, su uso es limitado en la práctica clínica dado el elevado número de pacientes con sospecha de ICFEP. De este modo, los criterios de diagnósticos para ICFEP deberían basarse principalmente en mediciones no invasivas. Como ninguna variable no invasiva por sí sola puede corroborar o refutar adecuadamente el diagnóstico, se han introducido diferentes combinaciones de parámetros clínicos, ecocardiográficos y/o bioquímicos. Los últimos años se han elaborado una gran cantidad de definiciones de ICFEP siendo las más conocidas y estudiadas la puntuación H_2_FPEF^8^ y el algoritmo HFA-PEFF de la Heart Failure Association^9^. Estas definiciones varían en su enfoque del diagnóstico, así como en su sensibilidad y especificidad y en el número y complejidad de las variables utilizadas para su cálculo. La puntuación H_2_FPEF es una herramienta basada en la evidencia derivada y validada en los pacientes remitidos para pruebas invasivas. Las variables incluidas en la puntuación H_2_FPEF son: obesidad (índice de masa corporal [IMC] ≥ 30 kg/m^2^; 2 puntos), fibrilación auricular (3 puntos), edad > 60 años (1 punto), tratamiento con ≥ 2 antihipertensivos (1 punto), la relación entre el flujo de entrada mitral en diástole temprana y las velocidades tisulares del anillo mitral (cociente E/e’ > 9; 1 punto) y estimación ecocardiográfica de la presión sistólica de la arteria pulmonar > 35 mmHg (1 punto). Se ha sugerido que una puntuación total de ≥ 6 puntos confirma el diagnóstico de ICFEP, mientras que una puntuación de 0 o 1 se sugiere para excluir el diagnostico^8^. El H_2_FPEF se basa predominantemente en perfiles clínicos, en sencillos parámetros ecocardiográficos y permite estratificar el riesgo de los pacientes^10^, ^11^. En estudios anteriores publicados por nuestro grupo, encontramos que más del 40% de los diagnósticos de IC pueden considerarse no adecuados^12^, y que aquellos pacientes en el los que no se dispone de datos ecocardiográficos en su historia clínica presentan mayor morbimortalidad^13^. Es preciso por lo tanto estandarizar el diagnóstico de IC para minimizar los diagnósticos erróneos^18^. La puntuación H_2_FPEF puede ser una herramienta útil en este proceso de mejora.

El objetivo de nuestro estudio es evaluar, en una cohorte de pacientes con diagnóstico de IC en atención primaria, la probabilidad de presentar ICFEP según el *score* H_2_FPEF, y analizar las características de los pacientes asociados a esta probabilidad.

Como objetivo secundario se pretende valorar la relación entre la morbimortalidad a un año con la puntuación del *score* y el resto de variables recogidos.

## Métodos

### Población de estudio

Pacientes con registro de IC (CIE-10: I.50) en la historia clínica informatizada de 7 equipos de atención primaria de la ciudad de Barcelona que atienden una población aproximada de 200.000 habitantes. Se incluyeron los pacientes con diagnóstico de IC en la historia clínica informatizada de AP (eCAP), que presentaban una fracción de eyección de ventrículo izquierdo (FEVI) ≥ 50%. Se excluyeron los participantes que no aceptaron participar y aquellos que tenían criterios ecocardiográficos de IC con FEVI reducida, recuperada o desconocida.

El objetivo del estudio es valorar la probabilidad diagnóstica de ICFEP en esta población según el *score* y como objetivo secundario, valorar la relación de esta probabilidad y del resto de variables recogidas con el objetivo combinado mortalidad por todas las causas u hospitalización por descompensación de IC

### Diseño

Estudio descriptivo transversal. El periodo de estudio fue de diciembre de 2023 a diciembre de 2024.

### Variables y recogida de datos

Se extrajeron listados de pacientes con diagnóstico de IC de los médicos participantes. Se recogió el consentimiento informado de los pacientes que cumplían con los criterios de inclusión. Se registraron las variables clínicas, las comorbilidades, las pruebas de laboratorio, los tratamientos, las variables ecocardiográficas y los eventos previos (ingresos en los últimos 5 años, y las urgencias hospitalarias en año previo) (tabla 1). Se definió complejidad según los criterios del programa de prevención y atención a la cronicidad del Departament de Salut de Catalunya, considerando como paciente crónico complejo (PCC) a aquel con multimorbilidad, fragilidad avanzada o una condición clínica que implica una gestión clínica difícil^14^. Se definió mal control en diabetes como una hemoglobina glicosilada (HbA1c) > 7% en los pacientes menores de 65 años y HbA1c > 8,5% en los pacientes mayores de 75 años, en línea con las recomendaciones de individualización de objetivos terapéuticos propuestas por la American Diabetes Association (ADA) y la Sociedad Española de Diabetes (SED)^15^, ^16^. La información se introdujo de forma codificada y se guardó en una base de datos protegida por contraseña en ordenadores del Instituto Catalán de la Salud.

**Tabla 1:** Características generales de la población de estudio estratificada por sexo

	Total	Mujeres	Varones	Valor de p global	N
	N = 545	N = 328	N = 217		
*Mortalidad por todas las causas*	54 (9,91%)	34 (10,4%)	20 (9,22%)	0,769	545
*Hospitalización por insuficiencia cardiaca*	104 (19,1%)	65 (19,8%)	39 (18,0%)	0,671	545
Evento combinado	142 (26,1%)	90 (27,4%)	52 (24,0%)	0,421	545
*Urgencias por insuficiencia cardiaca*	76 (13,9%)	54 (16,5%)	22 (10,1%)	0,050	545
*NT-ProBNP (pg/ml) (media, DE)*	3.315 (8798)	2.833 (3469)	4.004 (13077)	0,290	299
*Número antihipertensivos (media, DE)*	2,26 (1,13)	2,15 (1,10)	2,43 (1,15)	0,007	542
*Fracción de eyección % (media, DE)*	60,2 (6,42)	60,4 (6,34)	59,9 (6,55)	0,167	545
*PAPs (mmHg) (media, DE)*	32,7 (19,5)	33,9 (19,3)	30,9 (19,7)	0,156	362
*Cociente E/E’ (media, DE)*	11,7 (6,53)	12,5 (7,36)	10,8 (5,19)	0,059	205
*Edad (media, DE)*	82,3 (9,61)	83,8 (9,22)	80,2 (9,80)	< 0,001	545
*Colesterol total >* *200* *mg/dl*	123 (23,1%)	93 (28,7%)	30 (14,4%)	< 0,001	533
*Clase NYHA*					
I	95 (22,3%)	47 (18,4%)	48 (28,2%)	0,069	426
II	239 (56,1%)	147 (57,4%)	92 (54,1%)		
III-IV	92 (21,6%)	62 (24,2%)	30 (17,6%)		
*IMC (kg/m*^*2*^*) (media, DE)*	28,5 (5,56)	28,4 (5,82)	28,6 (5,17)	0,292	522
*Hb1Ac en diabetes (%) (media, DE)*	6,89 (1,24)	7,05 (1,26)	6,71 (1,19)	0,057	182
*TSH (mlU/l) (media, DE)*	2,83 (5,96)	2,70 (1,74)	3,02 (9,28)	0,036	520
*Hemoglobina (g/dl) (media, DE)*	13,2 (1,73)	12,9 (1,55)	13,6 (1,89)	< 0,001	536
*Ferritina (ng/ml) (media, DE)*	158 (200)	141 (171)	184 (239)	0,065	473
*Tabaco*					
No	438 (81,3%)	297 (91,4%)	141 (65,9%)	< 0,001	539
Exfumador	83 (15,4%)	21 (6,46%)	62 (29,0%)		
Sí	18 (3,34%)	7 (2,15%)	11 (5,14%)		
*Creatinina (mg/dl) (media, DE)*	1,11 (0,55)	1,00 (0,47)	1,29 (0,61)	< 0,001	537
*Filtrado glomerular (ml/min/1,73* *m*^*2*^*)*	58,6 (20,4)	58,3 (19,9)	59,1 (21,1)	0,659	536
*Potasio (mmol/l) (media, DE)*	4,33 (0,49)	4,30 (0,51)	4,38 (0,45)	0,031	539
*Microalbuminuria (mg/g) (media, DE)*	98,8 (348)	62,6 (224)	149 (464)	0,268	406
*Paciente crónico complejo*	218 (40,0%)	146 (44,5%)	72 (33,2%)	0,011	545
*Ictus*	81 (14,9%)	40 (12,2%)	41 (18,9%)	0,042	545
*Fibrilación auricular*	292 (53,6%)	182 (55,5%)	110 (50,7%)	0,312	545
*Anemia ferropénica*	73 (13,4%)	46 (14,0%)	27 (12,4%)	0,687	545
*Ansiedad*	144 (26,4%)	100 (30,5%)	44 (20,3%)	0,011	545
*Apnea del sueño*	79 (14,5%)	33 (10,1%)	46 (21,2%)	< 0,001	545
*Asma*	43 (7,89%)	30 (9,15%)	13 (5,99%)	0,240	545
*Atención domiciliaria*	124 (22,8%)	92 (28,0%)	32 (14,7%)	< 0,001	545
*Enfermedad coronaria*	134 (24,6%)	56 (17,1%)	78 (35,9%)	< 0,001	545
*Depresión*	112 (20,6%)	82 (25,0%)	30 (13,8%)	0,002	545
*Diabetes tipo 2*	179 (32,8%)	92 (28,0%)	87 (40,1%)	0,005	545
*Mal control en diabetes*	27 (15,3%)	16 (18,0%)	11 (12,6%)	0,440	176
*Hipercolesterolemia*	272 (49,9%)	159 (48,5%)	113 (52,1%)	0,462	545
*Gota*	61 (11,2%)	25 (7,62%)	36 (16,6%)	0,002	545
*Hipotiroidismo*	47 (8,62%)	39 (11,9%)	8 (3,69%)	0,001	545
*Hipertensión*	424 (77,8%)	252 (76,8%)	172 (79,3%)	0,573	545
*Mal control en hipertensión*	89 (21,0%)	46 (18,3%)	43 (25,0%)	0,125	423
*Enfermedad pulmonar obstructiva crónica*	102 (18,7%)	40 (12,2%)	62 (28,6%)	< 0,001	545
*Enfermedad arterial periférica*	60 (11,0%)	22 (6,71%)	38 (17,5%)	< 0,001	545
*Cáncer*	157 (28,8%)	83 (25,3%)	74 (34,1%)	0,034	545
*Obesidad*	245 (45,0%)	152 (46,3%)	93 (42,9%)	0,476	545
*Enfermedad renal crónica*	208 (38,2%)	121 (36,9%)	87 (40,1%)	0,507	545
*Antiinflamatorios no esteroideos*	21 (3,85%)	10 (3,05%)	11 (5,07%)	0,331	545
*Ansiolíticos e hipnóticos*	194 (35,6%)	132 (40,2%)	62 (28,6%)	0,007	545
*Antiagregantes y anticoagulantes*	420 (77,1%)	240 (73,2%)	180 (82,9%)	0,011	545
*Antidepresivos*	171 (31,4%)	125 (38,1%)	46 (21,2%)	< 0,001	545
*Psicotrópicos*	279 (51,2%)	189 (57,6%)	90 (41,5%)	< 0,001	545
*ISGLT2*	185 (33,9%)	93 (28,4%)	92 (42,4%)	0,001	545
*Hipolipemiantes*	296 (54,3%)	149 (45,4%)	147 (67,7%)	< 0,001	545
Antihipertensius_blocadors_alfa_binari	22 (4,04%)	5 (1,52%)	17 (7,83%)	0,001	545
*Bloqueantes beta*	310 (56,9%)	176 (53,7%)	134 (61,8%)	0,075	545
*Calcioantagonistas*	129 (23,7%)	71 (21,6%)	58 (26,7%)	0,206	545
*Diuréticos*	389 (71,4%)	247 (75,3%)	142 (65,4%)	0,016	545
*IECA o ARAII*	252 (46,2%)	149 (45,4%)	103 (47,5%)	0,704	545
*Fracción eyección registrada*	545 (100%)	328 (100%)	217 (100%)	—	545
*Score*_H_2_FPEF	4,60 (2,06)	4,66 (1,99)	4,52 (2,16)	0,337	545
*Score*_H_2_FPEF_group_2				0,172	545
Baixa o moderada probabilitat ICFEP	362 (66,4%)	210 (64,0%)	152 (70,0%)		
Alta probabilitat ICFEP	183 (33,6%)	118 (36,0%)	65 (30,0%)		
Tiempo_insuficiencia_cardiaca	5,02 (4,78)	5,41 (4,93)	4,45 (4,48)	0,023	530

ARAII: antagonistas de los receptores de angiotensina II; Cociente E/e’: relación entre el flujo de entrada mitral en diástole temprana y las velocidades tisulares del anillo mitral; CUAP: centro de urgencias de atención primaria; DE: desviación estándar; HbA1c: hemoglobina glicosilada; ICFEP: insuficiencia cardiaca con fracción de eyección preservada; IECA: inhibidores de la enzima convertidora de angiotensina; IMC: índice de masa corporal; ISGLT2: inhibidores del cotransportador sodio-glucosa tipo 2. Mal control en diabetes: HbA1c > 7% en los pacientes menores de 65 años y HbA1c > 8,5% en los pacientes mayores de 75 años. Mal control en hipertensión: presión arterial sistólica ≥ 140 y/o presión arterial diastólica ≥ 90 mmHg; PAPs: presión arterial pulmonar sistólica.

Con las variables registradas en eCAP se calculó el H_2_FPEF para cada uno de los pacientes. Se clasificaron los pacientes en: probabilidad alta (puntuación igual o mayor 6), probabilidad media (2-5) y diagnóstico no confirmado (0-1). Al año de la inclusión de los pacientes, mediante revisión eCAP y contacto telefónico se recogieron los eventos ocurridos (mortalidad por cualquier causa e ingresos por descompensación de IC).

### Análisis estadístico

Las variables categóricas se expresaron mediante frecuencias y porcentajes y las cuantitativas mediante media y desviación estándar. Se realizó un análisis bivariado para explorar la asociación entre la agrupación del *score* H_2_FPEF y las demás variables. Para las variables categóricas, se empleó la prueba de Chi-cuadrado. Se evaluó la normalidad de las variables cuantitativas mediante la prueba de Shapiro-Wilk. En función de los resultados obtenidos, se aplicaron pruebas t de Student o U de Mann-Whitney para comparaciones entre 2 grupos, y ANOVA o Kruskal-Wallis para más de 2 grupos.

También se llevó a cabo un análisis bivariado para identificar factores asociados al evento combinado, calculando los *odds ratio* (OR) con sus respectivos intervalos de confianza del 95%. Las variables que presentaron un valor de p < 0,10 en este análisis, se incorporaron como covariables en modelos de regresión logística multivariante, excluyendo aquellas usadas para calcular el *score* H2FPEF. Para evaluar el rendimiento del modelo, se calculó la curva ROC y el área bajo la curva (AUC).

El nivel de significación estadística se estableció en p < 0.05. Todos los análisis se realizaron con el *software* R versión 4.3.2.

### Tamaño muestral

Para la población de los CAPs participantes y considerando una prevalencia del 2% de IC (50% ICFEP), con un nivel de confianza del 95% (IC 95%) y una potencia estadística del 80% (α = 0,05; β = 0,20), la muestra representativa sería de 376 pacientes. La estimación incluyó una tasa de pérdidas anticipada del 10%.

## Resultados

### Características basales de la población

**Figura 1:**
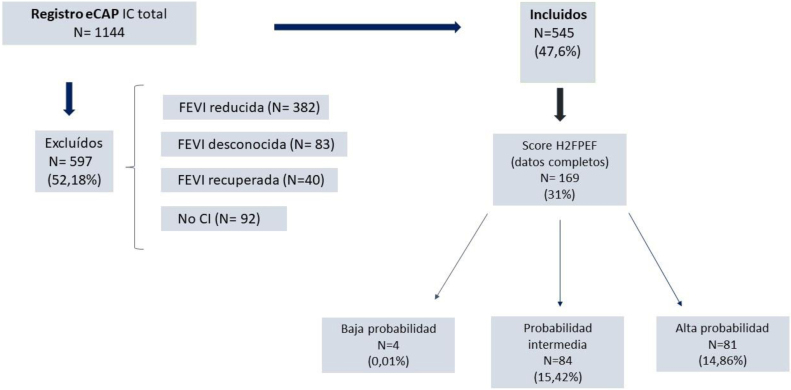
Diagrama de flujo del estudio. CI: consentimiento informado; eCAP: Historia clínica informatizada de atención primaria; FEVI: fracción de eyección del ventrículo izquierdo.

Se identificaron 1.144 pacientes con diagnóstico de IC en la historia clínica informatizada de los 7 centros de atención primaria participantes. Se excluyeron 597 (92 por falta de consentimiento, 382 por ICFER, 83 por fracción de eyección desconocida y 40 por fracción de eyección recuperada, incluyendo finalmente 545 pacientes. El diagrama de flujo del estudio se muestra en la figura 1.

Las características demográficas, clínicas y los tratamientos de los 545 pacientes incluidos en el estudio se muestran en la tabla 1, estratificadas por sexo. De los 545 pacientes incluidos, el 60,2% eran mujeres. Las mujeres con ICFEP eran significativamente de mayor edad, tenían una peor clase funcional según la NYHA y presentaban una mayor prevalencia de ansiedad, depresión e hipotiroidismo, y un peor control de las cifras de colesterol total. Asimismo, se observó una menor proporción de mujeres fumadoras, así como una menor prevalencia de diabetes mellitus, neoplasias, enfermedad coronaria, enfermedad pulmonar obstructiva crónica, apneas del sueño y enfermedad arterial periférica. Las mujeres también presentaron una mayor complejidad y precisaron mayor atención domiciliaria. En relación con los tratamientos, una mayor proporción de mujeres recibió diuréticos y psicofármacos, en cambio los hombres recibieron más antiagregantes y anticoagulantes, hipolipemiantes e inhibidores del cotransportador sodio-glucosa tipo 2.

Se pudo obtener información completa de todas las variables para realizar el *score* H_2_FPEF en 169 pacientes (26,9%). En este grupo, según el *score* H_2_FPEF, se encontró que el 2,3% tenían una baja probabilidad, el 49,7% tenían probabilidad media y el 47,9% una alta probabilidad de padecer una ICFEP.

Las características generales de los 169 pacientes en que se pudo calcular el *score* se muestran en la tabla 2. El grupo de mayor probabilidad de ICEP tenía mayor edad (84 años, DE: 10,1) y presentaba una mayor complejidad respecto a los grupos de probabilidad baja y media. También tenían mayor probabilidad de fibrilación auricular (93,8%), hipertensión (79%) y obesidad (63%) y presentaron una menor tasa de filtración glomerular (57 ml/min/1,73 m^2^, DE: 19,5). Las cifras del NT-ProBNP fueron superiores (3.000 pg/ml, DE: 4.230). Recibieron más antiagregantes y anticoagulantes (96,3%), diuréticos (90,1%) y betabloqueantes (72,8%) que los pacientes que presentaron una probabilidad baja o media de ICFEP según el *score*.

**Tabla 2:** Características generales de la población que presenta todas las variables para el cálculo del score H2FPEF

	Total	Baja probabilidad ICFEP	Moderada probabilidad ICFEP	Alta probabilidad ICFEP	Valor de p global	N
	N = 169	N = 4	N = 84	N = 81		
*Mortalidad por todas las causas*	13 (7,69%)	0 (0,00%)	4 (4,76%)	9 (11,1%)	0,332	169
*Hospitalización por insuficiencia cardiaca*	33 (19,5%)	0 (0,00%)	12 (14,3%)	21 (25,9%)	0,112	169
*Evento combinado*	45 (26,6%)	0 (0,00%)	16 (19,0%)	29 (35,8%)	0,027	169
*Urgencias por insuficiencia cardiaca*	13 (7,69%)	1 (25,0%)	4 (4,76%)	8 (9,88%)	0,148	169
*NT-ProBNP (pg/ml) (media, DE)*	2.593 (3.886)	218 (,)	2.216 (3.514)	3.003 (4.233)	0,023	118
*Número antihipertensivos (media, DE)*	2,10 (1,18)	0,50 (0,58)	1,82 (1,15)	2,47 (1,10)	< 0,001	169
*Fracción de eyección % (media, DE)*	60,6 (6,04)	58,0 (4,32)	60,5 (5,72)	60,9 (6,45)	0,683	169
*PAPs (mmHg) (media, DE)*	31,0 (19,6)	12,8 (14,8)	22,6 (18,8)	40,6 (15,8)	< 0,001	169
*Cociente E/E’ (media, DE)*	12,0 (7,00)	5,58 (2,63)	10,7 (6,60)	13,8 (7,11)	< 0,001	169
*Edad (media, DE)*	80,3 (9,86)	60,2 (16,3)	78,7 (10,1)	83,1 (7,60)	< 0,001	169
*Género (mujer):*	97 (57,4%)	2 (50,0%)	47 (56,0%)	48 (59,3%)	0,910	169
*Colesterol total >* *200 mg/dl*	37 (22,2%)	1 (25,0%)	23 (28,0%)	13 (16,0%)		
*Clase NYHA*						
I	32 (24,8%)	1 (50,0%)	16 (25,4%)	15 (23,4%)	0,875	129
II	72 (55,8%)	1 (50,0%)	36 (57,1%)	35 (54,7%)		
III-IV	25 (19,4%)	0 (0,00%)	11 (17,5%)	14 (21,9%)		
*IMC (kg/m*^*2*^*) (media, DE)*	29,3 (5,75)	27,6 (0,57)	28,8 (5,68)	29,9 (5,93)	0,318	169
*Hb1Ac en diabetes (%) (media, DE)*	6,82 (1,19)	6,20 (,)	6,97 (1,10)	6,70 (1,28)	0,442	60
*TSH (mlU/l) (media, DE)*	2,65 (1,80)	1,97 (1,21)	2,45 (1,76)	2,89 (1,85)	0,145	166
*Hemoglobina (g/dl) (media, DE)*	13,2 (1,80)	13,7 (0,98)	13,1 (1,93)	13,2 (1,69)	0,720	169
*Ferritina (ng/ml) (media, DE)*	190 (235)	51,0 (5,20)	183 (224)	204 (251)	0,205	147
*Tabaco*						
No	130 (77,4%)	2 (50,0%)	66 (79,5%)	62 (76,5%)	0,418	168
Exfumador	33 (19,6%)	2 (50,0%)	14 (16,9%)	17 (21,0%)		
Sí	5 (2,98%)	0 (0,00%)	3 (3,61%)	2 (2,47%)		
*Creatinina (mg/dl) (media, DE)*	1,09 (0,43)	0,90 (0,08)	1,08 (0,40)	1,11 (0,48)	0,677	169
*Filtrado glomerular (ml/min/1,73* *m*^*2*^*)*	59,9 (19,8)	79,1 (20,8)	61,9 (19,5)	57,0 (19,5)	0,032	169
*Potasio (mmol/l) (media, DE)*	4,29 (0,47)	4,38 (0,29)	4,40 (0,44)	4,18 (0,47)	0,012	168
*Microalbuminuria (mg/g) (media, DE)*	67,4 (203)	22,0 (,)	85,3 (270)	50,2 (102)	0,489	135
*Paciente crónico complejo*	51 (30,2%)	0 (0,00%)	17 (20,2%)	34 (42,0%)	0,004	169
*Ictus*	23 (13,6%)	0 (0,00%)	14 (16,7%)	9 (11,1%)	0,527	169
*Fibrilación auricular*	96 (56,8%)	0 (0,00%)	20 (23,8%)	76 (93,8%)	< 0,001	169
*Anemia ferropénica*	18 (10,7%)	0 (0,00%)	10 (11,9%)	8 (9,88%)	0,876	169
*Ansiedad*	42 (24,9%)	0 (0,00%)	24 (28,6%)	18 (22,2%)	0,406	169
*Apnea del sueño*	26 (15,4%)	0 (0,00%)	16 (19,0%)	10 (12,3%)	0,406	169
*Asma*	17 (10,1%)	1 (25,0%)	9 (10,7%)	7 (8,64%)	0,421	169
*Atención domiciliaria*	25 (14,8%)	0 (0,00%)	11 (13,1%)	14 (17,3%)	0,748	169
*Enfermedad coronaria*	41 (24,3%)	0 (0,00%)	20 (23,8%)	21 (25,9%)	0,727	169
*Depresión*	34 (20,1%)	0 (0,00%)	18 (21,4%)	16 (19,8%)	0,814	169
*Diabetes tipo 2*	56 (33,1%)	1 (25,0%)	25 (29,8%)	30 (37,0%)	0,585	169
*Mal control en diabetes*	10 (17,9%)	0 (0,00%)	7 (28,0%)	3 (10,0%)	0,309	56
*Hipercolesterolemia*	79 (46,7%)	3 (75,0%)	38 (45,2%)	38 (46,9%)	0,541	169
*Gota*	23 (13,6%)	0 (0,00%)	10 (11,9%)	13 (16,0%)	0,725	169
*Hipotiroidismo*	11 (6,51%)	1 (25,0%)	3 (3,57%)	7 (8,64%)	0,110	169
*Hipertensión*	123 (72,8%)	1 (25,0%)	58 (69,0%)	64 (79,0%)	0,032	169
*Mal control en hipertensión*	31 (25,2%)	0 (0,00%)	15 (25,9%)	16 (25,0%)	1,000	123
*Enfermedad pulmonar obstructiva crónica*	39 (23,1%)	1 (25,0%)	23 (27,4%)	15 (18,5%)	0,302	169
*Enfermedad arterial periférica*	13 (7,69%)	0 (0,00%)	8 (9,52%)	5 (6,17%)	0,686	169
*Cáncer*	47 (27,8%)	2 (50,0%)	19 (22,6%)	26 (32,1%)	0,186	169
*Obesidad*	90 (53,3%)	0 (0,00%)	39 (46,4%)	51 (63,0%)	0,007	169
*Enfermedad renal crónica*	59 (34,9%)	1 (25,0%)	26 (31,0%)	32 (39,5%)	0,529	169
*Antiinflamatorios no esteroideos*	7 (4,14%)	1 (25,0%)	2 (2,38%)	4 (4,94%)	0,085	169
*Ansiolíticos e hipnóticos*	42 (24,9%)	0 (0,00%)	21 (25,0%)	21 (25,9%)	0,730	169
*Antiagregantes y anticoagulantes*	137 (81,1%)	2 (50,0%)	57 (67,9%)	78 (96,3%)	< 0,001	169
*Antidepresivos*	48 (28,4%)	0 (0,00%)	24 (28,6%)	24 (29,6%)	0,707	169
*Psicotrópicos*	68 (40,2%)	0 (0,00%)	33 (39,3%)	35 (43,2%)	0,262	169
*ISGLT2*	62 (36,7%)	2 (50,0%)	32 (38,1%)	28 (34,6%)	0,699	169
*Hipolipemiantes*	100 (59,2%)	2 (50,0%)	45 (53,6%)	53 (65,4%)	0,250	169
*Bloqueantes beta*	98 (58,0%)	1 (25,0%)	38 (45,2%)	59 (72,8%)	< 0,001	169
*Calcioantagonistas*	38 (22,5%)	0 (0,00%)	17 (20,2%)	21 (25,9%)	0,532	169
*Diuréticos*	130 (76,9%)	0 (0,00%)	57 (67,9%)	73 (90,1%)	< 0,001	169
*IECA o ARAII*	81 (47,9%)	1 (25,0%)	39 (46,4%)	41 (50,6%)	0,658	169
*Fracción eyección registrada*	169 (100%)	4 (100%)	84 (100%)	81 (100%)		169
Score *H*_*2*_*FPEF (media, DE)*	5,34 (2,18)	0,50 (0,58)	3,75 (1,15)	7,23 (1,08)	< 0,001	169
*Tiempo_insuficiencia_cardiaca*	4,53 (4,74)	0,83 (0,30)	4,29 (4,19)	4,95 (5,27)	0,086	162

ARAII: antagonistas de los receptores de angiotensina II; Cociente E/e’: relación entre el flujo de entrada mitral en diástole temprana y las velocidades tisulares del anillo mitral; DE: desviación estándar; HbA1c: hemoglobina glicosilada; IECA: inhibidores de la enzima convertidora de angiotensina; IMC: índice de masa corporal; ISGLT2: inhibidores del cotransportador sodio-glucosa tipo 2; Mal control en diabetes: HbA1c > 7% en los pacientes menores de 65 años y HbA1c > 8,5% en los pacientes mayores de 75 años. Mal control en hipertensión: presión arterial sistólica ≥ 140 y/o presión arterial diastólica ≥ 90 mmHg. PAPs: presión arterial pulmonar sistólica.

### Morbimortalidad al año de la inclusión en relación al *score* H_2_FPEF

El análisis de morbimortalidad se llevó a cabo en el subgrupo de 169 pacientes en los que se disponía de todas las variables necesarias para el cálculo completo del *score* H_2_FPEF.

En el análisis bivariado, la incidencia del evento combinado (mortalidad por todas las causas y hospitalización por descompensación de IC) fue significativamente mayor en el grupo con alta probabilidad según el *score* H_2_FPEF ; OR: 2,49; IC 95%: 1,23-5,16; p = 0,01).

En la tabla 3 se presenta el análisis bivariado de todas las variables con el objetivo combinado (mortalidad por todas las causas y hospitalización por descompensación de IC).

**Tabla 3:** Análisis bivariado de las características de los pacientes para el evento combinado

	Total	No	Sí	OR	Valor de p ratio	N
	N = 169	N = 124	N = 45			
*Fracción de eyección (%) (media, DE)*	60,6 (6,04)	60,5 (5,96)	60,9 (6,32)	1,01 [0,96;1,07]	0,717	169
*Género (varón)*	72 (42,6%)	51 (41,1%)	21 (46,7%)	1,25 [0,62;2,50]	0,525	169
*Colesterol total >* *200* *mg/dl*	37 (22,2%)	27 (22,1%)	10 (22,2%)	1,01 [0,42;2,27]	0,977	
*Classe_NYHA*						
I	32 (24,8%)	25 (27,2%)	7 (18,9%)	Ref.	Ref.	129
II	72 (55,8%)	51 (55,4%)	21 (56,8%)	1,45 [0,56;4,15]	0,456	
III-IV	25 (19,4%)	16 (17,4%)	9 (24,3%)	1,98 [0,61;6,71]	0,260	
*Hb1Ac en diabetes (%) (media, DE)*	6,82 (1,19)	6,77 (1,11)	6,94 (1,40)	1,13 [0,71;1,80]	0,609	60
*TSH (mlU/l) (media, DE)*	2,65 (1,80)	2,67 (1,80)	2,59 (1,81)	0,97 [0,80;1,19]	0,796	166
*Hemoglobina (g/dl) (media, DE)*	13,2 (1,80)	13,3 (1,84)	12,8 (1,64)	0,86 [0,71;1,04]	0,111	169
*Ferritina (ng/ml) (media, DE)*	190 (235)	175 (219)	228 (271)	1,00 [1,00;1,00]	0,238	147
*Tabaco*						
No	130 (77,4%)	92 (74,8%)	38 (84,4%)	Ref.	Ref.	168
Exfumador	33 (19,6%)	27 (22,0%)	6 (13,3%)	0,55 [0,19;1,37]	0,208	
Sí	5 (2,98%)	4 (3,25%)	1 (2,22%)	0,67 [0,02;5,01]	0,726	
*Potasio (mmol/l) (media, DE)*	4,29 (0,47)	4,30 (0,47)	4,29 (0,45)	0,96 [0,46;1,99]	0,906	168
*Microalbuminuria (mg/g) (media, DE)*	67,4 (203)	75,1 (238)	49,8 (75,2)	1,00 [1,00;1,00]	0,528	135
*Paciente crónico complejo*	67 (39,6%)	44 (35,5%)	23 (51,1%)	1,89 [0,94;3,81]	0,072	169
*Ictus*	23 (13,6%)	15 (12,1%)	8 (17,8%)	1,58 [0,59;3,98]	0,354	169
*Fibrilación auricular*	18 (10,7%)	15 (12,1%)	3 (6,67%)	0,54 [0,12;1,77]	0,331	169
*Anemia ferropénica*	42 (24,9%)	27 (21,8%)	15 (33,3%)	1,79 [0,83;3,81]	0,136	169
*Ansiedad*	26 (15,4%)	23 (18,5%)	3 (6,67%)	0,33 [0,07;1,02]	0,054	169
*Apnea del sueño*	17 (10,1%)	14 (11,3%)	3 (6,67%)	0,58 [0,12;1,93]	0,402	169
*Asma*	25 (14,8%)	17 (13,7%)	8 (17,8%)	1,37 [0,52;3,39]	0,513	169
*Atención domiciliaria*	41 (24,3%)	29 (23,4%)	12 (26,7%)	1,20 [0,53;2,59]	0,658	169
*Enfermedad coronaria*	34 (20,1%)	26 (21,0%)	8 (17,8%)	0,82 [0,32;1,93]	0,667	169
*Depresión*	56 (33,1%)	40 (32,3%)	16 (35,6%)	1,16 [0,56;2,37]	0,687	169
*Mal control en diabetes*	10 (17,9%)	7 (17,5%)	3 (18,8%)	1,11 [0,20;4,85]	0,895	56
*Hipercolesterolemia*	79 (46,7%)	58 (46,8%)	21 (46,7%)	1,00 [0,50;1,98]	0,992	169
*Gota*	23 (13,6%)	20 (16,1%)	3 (6,67%)	0,39 [0,08;1,22]	0,112	169
*Hipotiroidismo*	11 (6,51%)	9 (7,26%)	2 (4,44%)	0,63 [0,08;2,62]	0,557	169
*Hipertensión*	123 (72,8%)	92 (74,2%)	31 (68,9%)	0,77 [0,37;1,67]	0,497	169
*Mal control en hipertensión*	31 (25,2%)	23 (25,0%)	8 (25,8%)	1,05 [0,39;2,63]	0,916	123
*Enfermedad pulmonar obstructiva crónica*	39 (23,1%)	29 (23,4%)	10 (22,2%)	0,94 [0,40;2,10]	0,889	169
*Enfermedad arterial periférica*	13 (7,69%)	8 (6,45%)	5 (11,1%)	1,82 [0,51;5,92]	0,337	169
*Cáncer*	47 (27,8%)	33 (26,6%)	14 (31,1%)	1,25 [0,58;2,62]	0,566	169
*Enfermedad renal crónica*	59 (34,9%)	44 (35,5%)	15 (33,3%)	0,91 [0,43;1,87]	0,805	169
*Antiinflamatorios no esteroideos*	7 (4,14%)	6 (4,84%)	1 (2,22%)	0,50 [0,02;3,15]	0,510	169
*Antiagregantes y anticoagulantes*	137 (81,1%)	95 (76,6%)	42 (93,3%)	4,07 [1,34;18,4]	0,011	169
*Psicofármacos*	68 (40,2%)	44 (35,5%)	24 (53,3%)	2,07 [1,03;4,17]	0,040	169
*ISGLT2*	62 (36,7%)	46 (37,1%)	16 (35,6%)	0,94 [0,45;1,90]	0,862	169
*Hipolipemiantes*	100 (59,2%)	77 (62,1%)	23 (51,1%)	0,64 [0,32;1,28]	0,207	169
*Fracción de eyección registrada*	169 (100%)	124 (100%)	45 (100%)	Ref.	Ref.	169
Score*_H*_*2*_*FPEF*	5,34 (2,18)	5,09 (2,17)	6,04 (2,08)	1,24 [1,05;1,46]	0,013	169
Score *H*_*2*_*FPEF baja/moderada ICFEP*	88 (52,1%)	72 (58,1%)	16 (35,6%)	Ref.	Ref.	169
Alta probabilidad ICFEP	81 (47,9%)	52 (41,9%)	29 (64,4%)	2,49 [1,23;5,16]	0,010	
*Tiempo_Insuficiencia_cardiaca*	4,53 (4,74)	4,61 (4,75)	4,33 (4,77)	0,99 [0,92;1,06]	0,740	162

DE: desviación estándar; HbA1c: hemoglobina glicosilada; ICFEP: insuficiencia cardiaca con fracción de eyección preservada; ISGLT2: inhibidores del cotransportador sodio-glucosa tipo 2; OR: *odds ratio*; TSH: hormona estimulante del tiroides.

**Figura 2:**
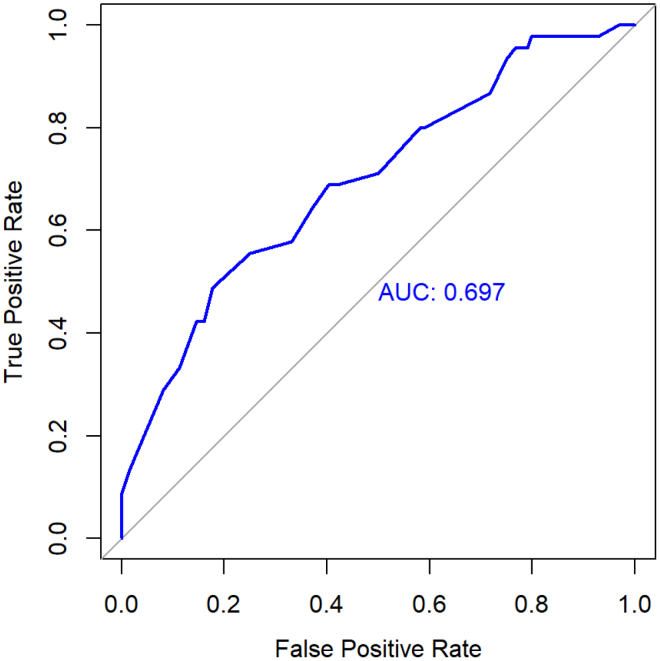
Curva ROC del modelo multivariado para el objetivo combinado

En el modelo multivariado ajustado por sexo, se incluyeron las variables que en el análisis bivariado presentaron una p < 0,10. En este análisis, una alta probabilidad de ICFEP según el *score* H_2_FPEF se asoció significativamente con un mayor riesgo del evento combinado (OR: 2,25; IC 95%: 1,08-4,78; p = 0,031). Además, el uso de psicofármacos también se asoció con un mayor riesgo (OR: 2,26; IC 95%: 1,05-4,95; p = 0,038) (tabla 4). El modelo mostró una curva ROC y el AUC de 0,697 (fig. 2).

**Tabla 4:** Análisis multivariado para el evento combinado en la población con todas las variables para el cálculo del *score* H_2_FPEF

Característica	OR	IC 95%	Valor de p
*Probabilidad ICFEP (score H*_*2*_*FPEF)*			
Baja o moderada	—	—	—
Alta	2,25	1,08-4,78	0,031
*Sexo*			
Mujer	—	—	—
Varón	2,13	0,97-4,81	0,063
*Uso de psicofármacos*			
No	—	—	—
Sí	2,26	1,05-4,95	0,038
*MACA o PCC*			
No	—	—	—
Sí	1,75	0,82-3,77	0,147
*Apnea del sueño*			
No	—	—	—
Sí	0,26	0,06-0,88	0,050

IC 95%: intervalo de confianza al 95%; OR: *odds ratio*.

*Variables incluidas en el *score* H_2_FPEF: obesidad (índice de masa corporal [IMC] ≥ 30 kg/m^2^), fibrilación auricular, edad > 60 años, tratamiento con ≥ 2 antihipertensivos, la relación entre el flujo de entrada mitral en diástole temprana y las velocidades tisulares del anillo mitral (cociente E/e’) > 9 y estimación ecocardiográfica de la presión sistólica de la arteria pulmonar > 35 mmHg.

## Discusión

Se obtuvo información completa de todas las variables para realizar el *score* H_2_FPEF en 169 pacientes (26,9%). En estos, encontramos que solo el 2,36% de los pacientes con un diagnóstico registrado de IC presentaba una baja probabilidad de ICFEP según el *score* H_2_FPEF.

Una alta probabilidad de ICFEP según el *score* H_2_FPEF se relacionó con una mayor morbimortalidad a un año (muerte por cualquier causa o ingreso por descompensación IC).

En los pacientes diagnosticados de IC en nuestra población, la proporción de diagnósticos de baja probabilidad fue inferior a la observada en estudios previos, en los que se ha reportó un rango que oscila entre el 17,3 y el 46,4%^13^, ^17^. Este hecho puede estar relacionado con una mejora en el diagnóstico y registro de la IC en atención primaria. Solo el registro de la fracción de eyección ha aumentado del 8,5 al 86,78% en los últimos años en nuestra área^12^, ^13^.

En el presente trabajo, los pacientes con mayor probabilidad de insuficiencia cardíaca con ICFEP según el *score* H_2_FPEF tenían una edad más avanzada, alta prevalencia de comorbilidades, además de las incluidas en el *score*, presentaba una peor tasa de filtración glomerular, coincidiendo con lo descrito en estudios previos en poblaciones occidentales^8^, ^9^, ^10^, ^17^, ^19^. No obstante, estudios recientes realizados en poblaciones asiáticas que evaluaron la utilidad del *score* H_2_FPEF han identificado fenotipos de pacientes con insuficiencia cardíaca con ICFEP caracterizados por una edad más joven y un menor índice de masa corporal^20^, ^21^, ^22^, ^23^, ^24^, ^26^, ^27^. En esta línea, estudios recientes analizaron el rendimiento del *score* H_2_FPEF en diferentes poblaciones asiáticas y propusieron nomogramas no invasivos específicos para su población, incorporando variables clínicas relevantes y ajustadas a dicho contexto, como la adaptación del punto de corte del IMC ≥ 25 kg/m^2^, de acuerdo con los criterios de obesidad en su población^24^, ^26^, ^27^. Estas adaptaciones metodológicas reflejan la necesidad de validar o ajustar el *score* según el entorno clínico y las características demográficas de la población objetivo.

Además, nuestra población mostró una mayor utilización de tratamientos dirigidos tanto a las comorbilidades cardiovasculares como no cardiovasculares, como el uso de antiagregantes y anticoagulantes, betabloqueantes, diuréticos, hipolipemiantes y psicofármacos.

En el análisis bivariado, los pacientes con una alta probabilidad de ICFEP según el *score* H_2_FPEF presentaron una incidencia significativamente mayor del evento combinado en comparación con aquellos con probabilidad baja o intermedia. Esta asociación se mantuvo en el análisis multivariado, donde una alta probabilidad de ICFEP según el *score* H_2_FPEF se asoció de forma independiente y significativa con una mayor probabilidad de presentar el evento combinado al año de seguimiento (OR: 2,25; IC 95%: 1,08-4,78; p = 0,031). Cabe destacar que, a diferencia de otros estudios donde esta relación solo se evidenció con seguimientos de entre 2 y 7,5 años^10^, ^20^, ^21^, ^22^, ^23^, ^24^, ^25^, ^27^, ^28^, en nuestra cohorte alcanzó significación estadística ya en el primer año. No obstante, en nuestro estudio no se realizó una curva ROC cuantitativa para establecer un punto de corte óptimo del *score*, ya que en el análisis multivariante el *score* H_2_FPEF cuantitativo no se relacionó significativamente con el evento combinado. Sería necesario diseñar estudios con mayor tamaño muestral y seguimiento más prolongado, para poder definir umbrales discriminativos y consolidar el uso del *score* H_2_FPEF como herramienta de estratificación pronóstica en el ámbito de la atención primaria.

Hasta la fecha, la mayoría de estudios que han evaluado el *score* H_2_FPEF se han realizado en el ámbito hospitalario y en poblaciones concretas, fundamentalmente asiáticas o en cohortes seleccionadas de ensayos clínicos, como el estudio ARIC, el estudio original de Reddy et al. o la cohorte TOPCAT, y los estudios que se han realizado en atención primaria no tienen suficiente potencia estadística al no alcanzar el tamaño muestral necesario^10^, ^20^, ^21^, ^22^, ^23^, ^24^, ^25^, ^26^, ^27^, ^28^, ^29^. Por su parte, Dal Canto et al., evaluaron la utilidad del *score* H_2_FPEF en un grupo de mujeres italianas con diabetes tipo 2 para detectar disfunción diastólica subclínica^30^.

Un estudio reciente de Laenens et al., evaluaron tanto el rendimiento diagnóstico como el valor pronóstico del *score* H_2_FPEF en una cohorte de pacientes ambulatorios derivados a Cardiología. Aunque representa una de las mayores series publicadas hasta la fecha en cuanto al tamaño muestral y tiempo de seguimiento, su aplicabilidad a la población atendida en atención primaria y en el contexto del sistema sanitario español sigue siendo incierta, lo que refuerza la necesidad de estudios como el presente^25^.

Se identificó una relación estadísticamente significativa entre el uso de psicofármacos, la morbimortalidad y puntuaciones elevadas en el *score* H_2_FPEF, sin que esto se observara con los diagnósticos clínicos de ansiedad o depresión. Dado que estudios previos han descrito una alta prevalencia de trastornos afectivos en los pacientes con IC, este hallazgo abre la puerta a futuras investigaciones^31^, ^32^.

El *score* H_2_FPEF destaca por su sencillez y rapidez, siempre que se disponga de las variables ecocardiográficas, ya que se basa en un número limitado de variables fácilmente accesibles, lo que lo convierte en una herramienta práctica para el cribado inicial. El *score* no incluye los péptidos natriuréticos, lo cual puede ser una ventaja, ya que además de no estar disponibles rutinariamente en algunos ámbitos de la atención primaria, diversos estudios han demostrado que sus niveles pueden ser bajos en los pacientes con ICFEP crónica^7^, ^18^, ^19^. De hecho, hasta un 20% de los pacientes con ICFEP pueden tener valores normales de péptidos^19^.

Dado que nuestro estudio demuestra la utilidad del *score* H_2_FPEF para la estratificación diagnóstica y su asociación con la morbimortalidad, sería recomendable que los informes ecocardiográficos incluyeran de forma sistemática todos los parámetros necesarios para calcular dicho *score*.

## Limitaciones del estudio y fortalezas

Las posibles pérdidas de información durante el seguimiento fueron previstas y contempladas en el cálculo del tamaño muestral.

No se pudo disponer de todos los parámetros ecocardiográficos necesarios para calcular el *score* H_2_FPEF en todos los pacientes. Para minimizar este sesgo, el análisis se centró en el subgrupo de pacientes con datos completos, garantizando así la validez interna de los resultados obtenidos en esa muestra. Esta limitación refleja una realidad frecuente en la práctica clínica, especialmente en atención primaria. No obstante, subraya la necesidad de mejorar el registro sistemático de estas variables en los informes ecocardiográficos, lo cual permitiría aplicar el *score* de forma más amplia y uniforme en el futuro. En este sentido, Saito et al. diseñaron y validaron un modelo de cribado simplificado sin variables ecocardiográficas, adaptado a las limitaciones de la atención primaria en Japón, lo que refuerza la utilidad de herramientas clínicas fácilmente aplicables en primera línea asistencial^27^.

Además, cabe señalar que las ecocardiografías no fueron realizadas por un único explorador, lo que podría introducir cierta variabilidad en la interpretación de los resultados. Asimismo, las determinaciones analíticas se recogieron en el contexto de la práctica asistencial habitual y no en condiciones centralizadas o homogéneas, lo que podría haber generado diferencias entre laboratorios.

Una limitación del estudio es el corto periodo de seguimiento, en comparación con los 2 a 7,5 años reportados en estudios previos, pese a ello nuestros hallazgos fueron consistentes con dichos trabajos y alcanzaron significación estadística ya en el primer año.

Una fortaleza de nuestro estudio fue la inclusión de centros de atención primaria localizados en distritos con diferentes niveles socioeconómicos, lo cual mejora la validez externa.

## Conclusiones

Nuestro estudio muestra que el uso del *score* H_2_FPEF en atención primaria es una herramienta útil tanto para mejorar el diagnóstico de la ICFEP como para estratificar el riesgo de morbimortalidad en esta población. Esto puede contribuir a reducir la variabilidad en la evaluación de los pacientes con sospecha de ICFEP en atención primaria y facilitar las decisiones clínicas.

**Lo conocido sobre el tema**

– La ICFEP representa el 50% de los casos de IC, y su diagnóstico es complejo en atención primaria.
– El *score* H_2_FPEF ayuda a estimar la probabilidad diagnóstica de la ICFEP.
– La utilidad en atención primaria ha sido poco estudiada.

**Qué aporta este estudio**

– Evalúa el *score* H_2_FPEF en los pacientes de atención primaria en España.
– Relaciona la alta probabilidad de la ICFEP con mayor morbimortalidad a un año.
– Propone su uso para mejorar el diagnóstico y pronóstico en atención primaria.

## Financiación

Este trabajo fue apoyado con una beca XB, dentro del Módulo de Investigación para profesionales de Atención Primaria del Institut Català de la Salut y Atenció Primària Barcelona Ciutat.

## Consideraciones éticas

Este estudio cumple con la Declaración de Helsinki y fue aprobado por el Comité de Ética en Investigación del Instituto de Investigación en Atención Primaria Jordi Gol (número de referencia 23/034-P). Se solicitó en consentimiento informado para participar en el estudio a los pacientes.

## Conflicto de intereses

Ninguno que declarar.
